# Psychedelic-Assisted Psychotherapy: When Two Traditions Meet

**DOI:** 10.1192/j.eurpsy.2022.1403

**Published:** 2022-09-01

**Authors:** E. Frecska, A. Kazai, P. Bokor

**Affiliations:** 1 University of Debrecen, Psychiatry Clinic, Debrecen, Hungary; 2 Multidisciplinary Association for the Research of Psychedelics, Psychology Group, Budapest, Hungary; 3 Karoli Gaspar University of the Reformed Church, Educational Psychology Group, Budapest, Hungary

**Keywords:** anthropology, psychedelics, Cultural diversity, Psychotherapy

## Abstract

**Introduction:**

After a long moratorium since the Controlled Substances Act was passed in 1970, there has been a resurgence of research on the potential therapeutic benefits of psychedelic (PE) compounds. It has been widely believed that the PE effect is a result of the interaction between the drug and the mindset of the patient (the “set”) with the external physical and social conditions (the “setting”). In order to control non-pharmacological variables and improve therapeutic outcome two types of psychological approaches to PE use have emerged traditionally. One is based on psychoanalytically informed talk therapy with low to moderate doses of a PE agent with the goal of facilitating a discharge of emotionally charged mental contents (psycholytic therapy). The other used one or several high doses of a PE to create an “overwhelming experience,” which was then followed up in integrative sessions (psychedelic therapy).

**Objectives:**

Currently, it is unclear which one is better than another, these two methods are frequently mixed, and all-together carry the name of psychedelic-assisted psychotherapy. There has also been some discrepancy about what is the right “set” and “setting”.

**Methods:**

To add some anchor points for (and at the same time warn about the limitations of ) the reemerging field of psychedelic-assisted psychotherapy the authors refer to anthropological observations in cultures, where PE use has a long practice historically.

**Results:**

As part of healing ceremonials PE has usually been administered in a tight community with shared cosmology (“set”) and ritual context (“setting”).

**Conclusions:**

These are difficult-to-reach conditions for someone coming from Western tradition.

**Disclosure:**

No significant relationships.

